# History of Spinal Cord “Pain” Pathways Including the Pathways Not Taken

**DOI:** 10.3389/fpain.2022.910954

**Published:** 2022-06-09

**Authors:** Allan Basbaum

**Affiliations:** Department of Anatomy, University of California San Francisco, San Francisco, CA, United States

**Keywords:** anterolateral quadrant, Brown-Séquard Syndrome, spinothalamic tract, multisynaptic spinal cord pathway, pain pathways

## Abstract

Traditional medical neuroanatomy/neurobiology textbooks teach that pain is generated by several ascending pathways that course in the anterolateral quadrant of the spinal cord, including the spinothalamic, spinoreticular and spinoparabrachial tracts. The textbooks also teach, building upon the mid-19th century report of Brown-Séquard, that unilateral cordotomy, namely section of the anterolateral quadrant, leads to contralateral loss of pain (and temperature). In many respects, however, this simple relationship has not held up. Most importantly, pain almost always returns after cordotomy, indicating that activation of these so-called “pain” pathways may be sufficient to generate pain, but they are not necessary. Indeed, Brown-Séquard, based on his own studies, eventually came to the same conclusion. But his new view of “pain” pathways was largely ignored, and certainly did not forestall Spiller and Martin's 1912 introduction of cordotomy to treat patients. This manuscript reviews the history of “pain” pathways that followed from the first description of the Brown-Séquard Syndrome and concludes with a discussion of multisynaptic spinal cord ascending circuits. The latter, in addition to the traditional oligosynaptic “pain” pathways, may be critical to the transmission of “pain” messages, not only in the intact spinal cord but also particularly after injury.

Noordenbos (1) “It is abundantly clear that an anterolateral or medullary tractotomy is anything but a mere interruption of the “pathway for pain”

Livingston (2) “In the treatment of pain there should be more physiologic means for its control than mere interruption of its communication”

## Introduction

Several years ago, I was invited to lecture on “The History of Neuropathic Pain” at the Berlin meeting of the Neuropathic Pain Special Interest Group. I assumed that I was chosen to present this lecture because most folks in the audience were born before any of the history took place. Interestingly, the word neuropathic did not enter the neurology literature until the latter half of the 20th century. Rather, the existence of nerve injury-induced pain was first recognized by Weir Mitchell during the American Civil War (No, I did not know Weir Mitchell personally). Of course, history does have a tendency to repeat itself, which I presume is what led to my invitation to write this piece, in essence on the History of the Pain Pathway, or to be more inviting, focusing according to our editor, Tony Yaksh, on pivotal moments that defined the Pain Pathway. This is an interesting and important topic, and as someone who believes that “The Bane of Pain is Plainly in the Brain” (3), the topic is a difficult one. Of course, there is no pain pathway, but rather neuroanatomical routes through which inputs reach the brain and lead to a pain percept. The question is whether the traditional “pain” pathways are necessary for input transmitted from the spinal cord to be experienced as pain.

**Figure 1:**
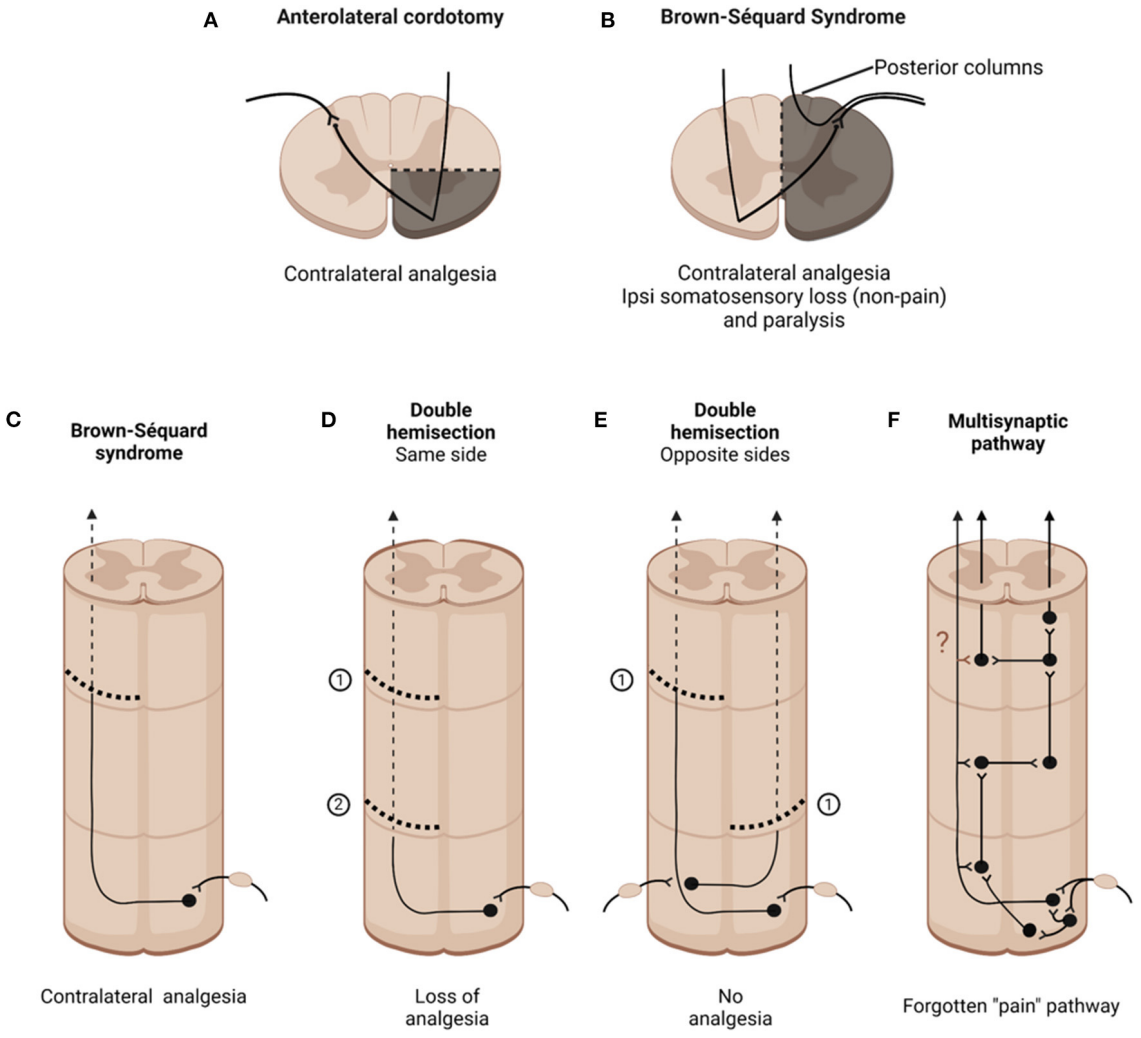
Consequences of cutting different spinal cord pathways. **(A)** Anterolateral cordotomy purportedly provokes contralateral analgesia below the lesion, albeit not permanently. **(B)** Brown-Sequard Syndrome produced by hemisection of the spinal cord. **(C–F)** Longitudinal views of different spinal cord pthways and lesions. **(C)** Unilateral hemisection; **(D)** Double hemisection performed on the same side of the spinal cord; **(E)** Spaced hemisection performed on opposite sides of the spinal cord; **(F)** Illustrates how a multisynaptic system that interconnects the two sides of the spinal cord can “escape” lesions to the long ascending pathways.

With these caveats in mind, I will take readers through a review of the association of different ascending pathways that course in the anterolateral quadrant to the generation of pain. I was helped significantly by colleagues as well as by a very detailed historical record described in a wonderful book by Lenz et al. (4). The story begins in the 19th century, with the preclinical observations of the Mauritian neurologist Brown-Séquard, who reported that hemisection of the spinal cord produced contralateral loss of pain and temperature [(5); Figures 1B,C]. These laboratory findings were followed by striking clinical observations, notably Brown-Séquard's 1862 report to the British Medical Association, in which he described a sailor who had been stabbed in the neck. Eight years after the stabbing, the sailor presented with paralysis and loss of non-painful tactile sensation ipsilateral to the knife entry and contralateral loss of pain and temperature. Here was the classic Brown-Séquard Syndrome, in which “pain” messages are transmitted by a pathway that crosses in the spinal cord. Also worth noting, but not commonly reported, is that his description of the syndrome that carries his name also highlighted the presence of ipsilateral symptoms, including hyperesthesia and pain.

### Pathways in the Anterolateral Quadrant Implicated in Pain

Subsequent studies narrowed down the spinal cord pathways that carry information leading to pain. These were comprehensively described in Gowers' 1886 two volume compendium (6), “Manual of Diseases of the Nervous System.” Gowers illustrated the pathology following the loss of pain in a patient whose upper cervical region was damaged by a bullet-generated bone fragment. Based on his observation that “the chief injury is clearly to the lateral column and gray matter, the posterior column being merely swollen, apparently by oedema,” Gowers concluded that “pain” is transmitted by a crossed pathway that courses in the anterolateral quadrant (Figure 1A). He wrote “We may accept as certain the fact that the tactile or painful impulse we feel crosses the middle line soon after entering the cord.″ Soon after these findings were published, William Spiller identified a patient with a bilateral tuberculoma of the anterolateral quadrant (7). Based on his finding that the patient experienced no pain below the spinal cord damage Spiller convinced the neurosurgeon, Edward Martin to perform the first anterolateral cordotomy for the relief of pain (8). In their post-operative report, they wrote: “The division of each anterolateral column was performed by Dr. Martin, Jan. 10, 1911. On January 22, there was great relief of pain in both lower limbs. The patient appeared very grateful for the relief from suffering and received only 1/0 grain morphine on the day following the operation and a similar amount 2 days later, and this was given for the pain caused by the operation.” The rest, as they say, is history.

### What Exactly Is cut by Anterolateral Cordotomy?

Interestingly, detailed studies of the anatomical basis of the pain relief from anterolateral cordotomy only came many years later, by Otfrid Foerster (9), a neurosurgeon with a remarkable pedigree, having studied with Karl Wernicke, Joseph Dejerine and Joseph Babinski. From 7 cases of pain relief after a cord lesion, he concluded that the relevant nerve fibers ascend less than two segments from their cell bodies before crossing. Furthermore, a poor result occurred when the lesion was not sufficiently deep, i.e., the incision did not completely transect the anterolateral quadrant. In another seminal postmortem finding in patients who underwent cordotomy for pain, Foerster and Gagel (10) identified retrograde degeneration of neurons in the contralateral dorsal horn, including large neurons in the most superficial dorsal horn. Subsequent neuroanatomical studies of the major ascending pathways that course in the anterolateral quadrant including spinothalamic and spinoreticular pathways, led to the conclusion that these pathways carry nociceptive messages to the brain, i.e., they are the “pain” pathways. Of course, the functional association of these ascending pathways with pain processing has been supported by numerous electrophysiological studies of antidromically activated, nociresponsive dorsal horn projection neurons in laminae I, V and VII (11–13). More recently, studies of the spinoparabrachial pathway (14, 15), which more selectively targets limbic brain regions involved in the affective component of the pain experience, have dominated preclinical research into ascending “pain” transmission systems.

The focus on these major anterolateral quadrant ascending pathways is understandable, and activation of these pathways does appear sufficient to generate pain. But which pathway actually carries the input that generates pain? And which brain areas engaged by the pathway are critical? Here the studies of William (Bill) Mehler, a student of Walle Nauta, who developed the silver staining methods that greatly increased the ability to detect degenerating fibers, are incredibly relevant. Mehler published the most beautiful and most comprehensive descriptions of the supraspinal targets where degenerating terminals are located after cordotomy (16, 17). His images are memorable, not only for their accuracy, but in my opinion, his hand drawn sketches of the different brainstem and thalamic targets where he identified degenerating fibers after cordotomy, in monkey, gorilla and human, have no equal in the neuroanatomical literature. Mehler clearly demonstrated that the patterns of degeneration following cordotomy include much more than the terminal targets of the spinothalamic, spinoreticular and spinoparabrachial pathways. Specifically, although the names of the pathways derive from the origin and their terminal targets, e.g., spinothalamic, each pathway collateralizes extensively, with terminations throughout the brainstem and even hypothalamus (18).

### How Effective Is Anterolateral Cordotomy for the Relief of Pain?

After the Spiller and Martin report, anterolateral cordotomy became rather commonplace for chronic pain management, even in non-terminal cancer pain patients. What is puzzling is that despite considerable evidence and concern that pathways that course in the anterolateral quadrant are neither the sole, nor even a necessary route through which pain is generated, the procedure continued to be performed. Cordotomy does produce pain relief contralateral to the lesion, but rarely, if ever, produces long lasting pain relief. For example, Cowley and Hitchcock (19) reported that pain relief after cordotomy for non-malignant disease fell to 20% within 1 year of the surgery, despite autopsy confirmation that the surgery was successful. In fact, the reappearance of pain is now the reason why the procedure is no longer recommended for non-terminal patients. Also remarkable is the report of Noordenbos and Wall (20) that somatosensory, including pain, and motor function, including activation of the cortex, can persist despite severe cord injury that spares only a sliver of one anterolateral quadrant.

Of particular interest is that recurrence of pain after cordotomy often does not appear in the region of the body where the pain that precipitated the surgery occurred. Peter Nathan, with whom, as a postdoc, I saw patients at the National Hospital for Neurology and Neurosurgery at Queen Square in London, elegantly discussed the recurrence of pains. In a 1956 article (21), he wrote “After the spinothalamic tract has been cut in man, loss of pain and temperature caudal to the lesion is expected. In a small proportion of patients, although analgesia in the usual sense of the term is present, painful or thermal stimuli applied to parts of the body caudal to the lesion arouse a sensation, which is felt, not at the place actually stimulated, but in a normally innervated part of the body.” He went on to write that “There may be a reference from the analgesic side to the analogous place on the opposite side of the body, but cranially, caudally, and in more complicated ways.” In effect, Nathan is describing a maladaptive reorganization of the “pain pathway” *after it was cut*!

Besides the recurrence of pain, there are numerous adverse effects associated with anterolateral cordotomy, including post-cordotomy dysesthesia, a severe central pain condition, potentially more severe than the pain that precipitated the cordotomy (22) and allochiria, namely the appearance of pain in the mirror region of the body, a condition described in the 19th century by Obersteiner (23). Also of interest is the report that post-stroke pain, yet another central (neuropathic) pain condition, occurs when the brain lesion clearly involves the spinothalamic tract (22, 24). And, of course, there is the Dejerine-Roussy syndrome (25), a devastating central neuropathic pain condition that results from damage to the thalamic target of the spinothalamic tract. That syndrome, of course, is precisely the opposite of what one would predict if damage interfered with the major target of the “pain pathway.” Apparently, although transient loss of pain does occur after cordotomy, over time, destruction of these pathways can trigger CNS changes that lead to devastating central neuropathic pain states, including chronic pain that can be provoked by normally non painful stimulation. Clearly, the traditional “pain” pathways are not necessary for pain to be generated.

From an historical perspective it is of particular interest that the concerns about a “pain pathway” in the anterolateral quadrant were raised by Brown-Séquard, almost two decades before the first cordotomy was performed. Specifically, despite Gowers' apparent confirmation of Brown-Séquard's earliest conclusions as to the contralateral transmission of “pain” information in the anterolateral quadrant, in a three-page paper published in 1894, the year that he died, Brown-Séquard rather emphatically rejected his own conclusions concerning the contribution of a crossed pain pathway (26). His new view followed upon an 1892 report of Frederick Mott, that lateral hemisection of the spinal cord in monkeys did not produce contralateral analgesia (27). In his own preclinical studies, Brown-Sequard found that a second hemisection performed caudal to the first one could reverse the contralateral analgesia produced by the first lesion (Figure 1D). Even slight stretch of the contralateral sciatic nerve could reinstate the pain that had been blocked by the hemisection. As beautifully summarized in a 2017 biography, Michael Aminoff (28) wrote that Brown-Séquard “now believed that the analgesia and hyperaesthesia related to inhibitory and excitatory effects, respectively, on central sensory mechanisms by the various interventions, rather than simply from the interruption of hard-wired pathways.” Brown-Séquard believed that the nervous system was best regarded as a system of dynamic processes rather than consisting of hard-wired structures. Aminoff concluded, rather sadly, that most neurologists failed to understand what Brown-Séquard had grasped so intuitively. Rather, they ignored Brown-Séquard's later observations and have chosen instead to cling to the earlier concept that he advanced and later repudiated.

Studies many years later provided further support for Brown-Séquard's new view. Derek Denny-Brown (29), a brilliant neurologist performed double hemisection studies (in his studies the second hemisection was performed rostral to the first) and found that the contralateral analgesia produced by the first hemisection was lost. Denny-Brown proposed that the second hemisection may have eliminated brainstem-derived descending inhibitory controls that are normally engaged by the first hemisection. The loss of descending controls, he proposed, sensitizes spinal cord nociceptive circuits. These findings lead to the conclusion that pain is not only a product of activation of projection neurons at the origin of the long ascending pathways in the anterolateral quadrant, but is also a product of maladaptive CNS circuit changes that can occur, particularly after damage to the “pain” pathways. Of course, studies of descending and other controls on spinal cord nociceptive processing came many years later.

### What Spinal Cord Pathways Transmit Pain-Provoking Information After Unilateral Anterolateral Cordotomy?

The post cordotomy return of pain poses two obvious and important questions: First, how can anterolateral cordotomy cut “*the* pain pathway” if pain returns? Second, what pathway or pathways underlie the return of the pain? The focus these days, at least in rodent studies, has been on dorsal horn projection neurons, but the very different receptive field properties of lamina VII spinoreticular neurons may be particularly relevant (30). Not only do spinoreticular neurons in laminae VII have large, often bilateral receptive fields, but they can also be antidromically activated from both ipsilateral and contralateral brainstem. In other words, the information that these neurons transmit could readily bypass a unilateral cordotomy. Furthermore, these neurons have exceptionally large inhibitory receptive fields (12), which if altered after spinal cord injury, could dramatically increase the transmission of “pain” messages to the brain. Lastly, but generally not discussed in reports on the transmission of somatic “pain” messages, is the contribution of the posterior columns to visceral pain (31).

### Multisynaptic Ascending “Pain” Pathways: The Forgotten Pathways

The last paragraph discusses how more traditional “pain” pathways could underlie the failures of cordotomy, but in another key paper Nathan (32) concluded that normal pain channels, namely the spinothalamic and related tracts, are indeed blocked by the cordotomy, but that afferent input eventually reaches consciousness via other routes, including some that course ipsilaterally. He postulated that propriospinal circuits, namely relatively long range, multisynaptic routes within the spinal cord, can transmit information from the spinal cord to the brain. There is, in fact a long, largely ignored, history demonstrating that a multisynaptic system of short neurons can transmit nociceptive information from the spinal cord to the brain. For the record, my PhD thesis research in the rat reported that the presence of spaced hemisections, one on the left side of the cord, and another several segments caudally on the right side, did not eliminate the ability of the animal to respond to a noxious stimulus [(33); Figure 1E]. As the rats could not respond with limb movements, they reported awareness of a noxious stimulus to the hindpaw by initiating a head movement to terminate the stimulus. Sadly, and this is a lesson to all young pain researchers, I soon learned that Karplus and Kriedl (34) had performed this experiment, in the cat, in the early 20^th^ century, with the same result. In fact, many other studies that preceded my thesis report suggested that short chains of neurons could relay pain relevant information from the cord to the brain. Looking at the cup half full, perhaps I could conclude that these authors “pre-confirmed” my results. Despite these many reports, with the increasing use of cordotomy, it was generally assumed that the post-operative loss of pain contralateral to the lesion resulted from cutting a “pain pathway,” namely the spinothalamic and spinoreticular tracts. That pain returned was largely ignored, as was a possible contribution of a multisynaptic pathway that escaped the cordotomy (Figure 1F).

The most detailed description of a multisynaptic spinal cord “pathway” can be found in a remarkable book, “Pain,” written by William Noordenbos, a neurosurgeon from the Netherlands (1). Noordenbos cites studies of Fajersztajn (35) who concluded that this system of neurons is “intimately mixed with the long ascending tracts, so the “pure” pain fiber tracts do not occur.” Cordotomy might concurrently cut into this system, however, unlike the long tracts, a single lesion would rarely, if ever, completely interrupt transmission of information. In addition to reviewing the arguments against there being a straight through “oligosynaptic pain” pathway, with few synaptic connections, Noordenbos illustrated numerous examples how *de novo* activity in an ascending multisynaptic system could underlie a host of chronic clinical pain conditions, from causalgia (the original term for burning pain produced by peripheral nerve damage) to post-herpetic neuralgia, post cordotomy girdle pain and even to phantom limb pain.

### Pain Pathways in the 21st Century

Is there any relevance to “reintroducing” a multisynaptic pathway to contemporary preclinical pain research scientists, who unquestionably focus on the long ascending “oligosynaptic” pathways that originate from the major spinal cord projection neurons? Unquestionably, there are monosynaptic and polysynaptic inputs from the periphery to these projection neurons, which contribute to the acute pain experience. There is also an enormous literature on the contribution, in the setting of tissue or nerve injury, of dorsal horn interneurons that enhance the input to the major projection neurons, via central sensitization processes (36). This includes loss of local and descending inhibitory controls and even engagement of rostral ventral medulla-derived facilitation of dorsal horn circuits (37). But do these interneurons only exert their effects on the projection neurons, or can they also access the brain via complex multisynaptic circuits? Perhaps normally “silent synapses” that are engaged after injury are at the origin of these multisynaptic pathways (38). Interestingly, that possibility was hypothesized by Gowers in the late 19^th^ century, when he wrote that “it is probable that some impulses that we do not feel do not cross. We must never forget that there is strong reason to believe that only a minority of the impulses that traverse afferent nerves affect our consciousness.”

Do these multisynaptic pathways also underlie the fact that unilateral injury can result in bilateral engagement of spinal cord nociceptive circuits, a finding with an important clinical correlate, namely in the appearance of contralateral pains in patient with CRPS, or in the induction of allochiria. Or, perhaps this pathway underlies the reappearance of pain after unilateral hemisection by a hemisection made caudal to the first one, which Brown-Séquard observed. Do collaterals of the projection neurons, along their ascending course to the brain, engage short chain multisynaptic circuits within the cord, taking a route that could not only bypass the hemisections but engage circuits both ipsilateral and contralateral to the lesions? That hypothesis is a central theme in the Noordenbos book. There is certainly evidence that collaterals of lamina I neurons engage local circuits at the level of the cell body, but Szucs et al. (39) concluded that axons of lamina I neurons also “govern large numbers of neurons, providing anatomical substrate for rostrocaudal “processing units” in the dorsal horn.” An interesting, and testable hypothesis, is that cordotomy (or hemisection) induces sprouting of the collaterals, resulting in the formation of new circuits, both ipsilateral and contralateral to the lesion (question mark in Figure 1F). Such new connections could greatly expand the networks with which the projection neurons are involved and could underlie the many pathophysiological consequences of cordotomy, including the loss of analgesia and the appearance of hyperesthetic states characteristic of many central pain conditions.

## Conclusion

What then is the significance of this historical review of the pain pathway? Yes, there are “pain” pathways in the anterolateral quadrant, activation of which, in the normal setting, generate pain experienced on the contralateral side of the body. On the other hand, although activation of these anterolateral quadrant located pathways are sufficient to generate pain, their activation is clearly not necessary. In fact, in the setting of tissue or nerve injury, there appear to be many complex ascending circuits, including multisynaptic ones, that can transmit the information that generates pain. These considerations provide a very different view of spinal cord “pain” pathways, and importantly, may be relevant to the development of novel pharmacotherapeutic approaches to ameliorate pain, not only by targeting the projection neurons at the origin of the major ascending “pain” pathways, but also by interfering with the interactions between the projection neurons and what to date are poorly studied multisynaptic spinal cord “pain” pathways. To paraphrase Livingston (2), there must be a more effective approach to chronic pain management than cutting illusive “pain” pathways.

## Author Contributions

The author confirms being the sole contributor of this work and has approved it for publication.

## Funding

This work was supported by a grant from the NIH R35 NS097306 and Open Philanthropy.

## Conflict of Interest

The author declares that the research was conducted in the absence of any commercial or financial relationships that could be construed as a potential conflict of interest. The handling editor TY and the reviewer HF declared a shared affiliation with the author at the time of review.

## Publisher's Note

All claims expressed in this article are solely those of the authors and do not necessarily represent those of their affiliated organizations, or those of the publisher, the editors and the reviewers. Any product that may be evaluated in this article, or claim that may be made by its manufacturer, is not guaranteed or endorsed by the publisher.

## References

[1] Noordenbos W. Pain: Problems Pertaining to the Transmission of Nerve Impulses Which Give Rise to Pain. Amsterdam: Elsevier (1959) p. 182.

[2] Livingston WK. Pain and Suffering, editors. Howard Fields. Washington, DC: IASP Press (1943) p. 250.

[3] Basbaum AI. The bane of pain is mainly in the brain. In: Linden DJ, editor. Think Tank: Forty Neuroscientists Explore the Biological Roots of Human Experience. New Haven, Conn: Yale University Press (2018). p. 128–134.

[4] Lenz FA Casey KL Jones EG Willis WD. The Human Pain System: Experimental and Clinical Perspective. Cambridge UK: Cambridge University Press (2010). p. 638.

[5] Brown-Séquard CE. Course of Lectures on the Physiology and Pathology of the Central Nervous System. Collins, Philadelphia: Collins, Printer (1860).

[6] Gowers WR. Notes on the diseases of the nervous system. II On the antero-lateral ascending tract of the spinal cord. Lancet. (1886) 1:1153–4. 10.1016/S0140-6736(00)49360-8

[7] Spiller WG. The occasional clinical resemblance between caries of the vertebrae and lumbothoracic syringomyelia, and the location within the spinal cord of the fibres for the sensations of pain and temperature. Univ Penn Med Bull. (1905) 18:147–1

[8] Spiller W Martin E. The treatment of persistent pain of organic origin in the lower part of the body by division of the anterolateral column of the spinal cord. JAMA. (1912) 58:489–90. 10.1001/jama.1912.0426005016500125996397

[9] Foerster O. Symptomatologie der Erkrankungen des Rückenmarks und seiner Wurzeln. in Handbuch der Neurologie. Bumke O, Foerster O. editors, (1936) p. 1–403. Berlin: Springer.

[10] Foerster O Gagel O. Die Vorderseitenstrangdurchschneidung beim Menschen. Eine klinisch-patho-physiologisch-anatomische Studie. Z Ges Neurol Psychiatr. (1932) 138: 1–92 10.1007/BF0287056334645050

[11] Andrew D Craig AD. 2002 Responses of spinothalamic lamina I neurons to maintained noxious mechanical stimulation in the caat. J Neurophysiol. (2002) 87:1889–91. 10.1152/jn.00577.2001 11929909

[12] Fields HL Clanton CH Anderson SD. Somatosensory properties of spinoreticular neurons in the cat. Brain Res. (1977) 120:49–66. 10.1016/0006-8993(77)90497-8 832119

[13] Willis WD Trevino DL Coulter JD Maunz RA. Responses of primate spinothalamic tract neurons to natural stimulation of hindlimb. J Neurophysiol. (1974) 37: 358–72. 10.1152/jn.1974.37.2.358 4205568

[14] Bernard JF Besson JM. The spino(trigemino)pontoamygdaloid pathway: electrophysiological evidence for an involvement in pain processes. J Neurophysiol. (1990) 63:473–90. 10.1152/jn.1990.63.3.473 2329357

[15] Todd AJ. Neuronal circuitry for pain processing in the dorsal horn. Nat Neurosci. (2010) 11:9823–836. 10.1038/nrn2947 21068766 PMC3277941

[16] Mehler W. R. The anatomy of the so-called pain tract in man: an analysis of the course and distribution of the ascending fibers of the fasciculus anterolateralis. In: Basic Research in Paraplegia. French J D, Porter RW, editors. Springfield: C.C. Thomas (1962). pp. 26–55

[17] Mehler WR Freeman ME Nauta WJ. Brain ascending axon degeneration following anterolateral cordotomy. an experiment study in the monkey. Brain. (1960) 83 718–50 10.1093/brain/83.4.718 13768983

[18] Zhang X Wenk HN Gokin AP Honda CN Giesler GJ Jr. Physiological studies of spinohypothalamic tract neurons in the lumbar enlargement of monkeys. J Neurophysiol. (1999) 82:1054–8. 10.1152/jn.1999.82.2.1054 10444696

[19] Cowie RA Hitchcock ER. 1982. The late results of antero-lateral cordotomy for pain relief. Acta Neurochir (Wien) 64: 39–50. 10.1007/BF01405617 6181657

[20] Noordenbos W Wall PD. Diverse sensory functions with an almost totally divided spinal cord. a case of spinal cord transection with preservation of part of one anterolateral quadrant. Pain. (1976) 2:185–95. 10.1016/0304-3959(76)90114-7 1026901

[21] Nathan PW. Reference of sensation at the spinal level J Neurol Neurosurg Psych. (1956) 19:88—. 10.1136/jnnp.19.2.88 13346381 PMC497190

[22] Cassinari V Pagni CA. Central Pain. London, UK: Oxford University Press (1969). p. 192.

[23] Obersteiner H. On allochiria: a peculiar sensory disorder. Brain. (1881) 4:153–63. 10.1093/brain/4.2.153

[24] Holmgren H Leijon G Boivie J Ilievska L. Central post-stroke pain–somatosensory evoked potentials in relation to location of the lesion and sensory signs. Pain. (1990) 40:43–52. 10.1016/0304-3959(90)91049-O 2339015

[25] Dejerine-Rousy J Roussey G. Le syndrome thalamique. Rev Neurol. (1906) 14:521–32

[26] Brown-Sequard CE. Remarques à propos des recherches du Dr. FW Mott sur les effets de la section d'une moitié latérale de la moelle épinière. Arch Physiol Norm Pathol. (1894) 6:195–8.

[27] Mott FW. Results of hemisection of the spinal cord in monkeys. Philos Trans R Soc Lond B. (1892) 183:1–59. 10.1098/rstb.1892.0001

[28] Aminoff MJ. The life and legacy of brown-séquard. Brain. (2017) 140:1525–32. 10.1093/brain/awx071 29050371

[29] Denny-Brown D. The enigma of crossed sensory loss with cord hemisection. In: Bonica JJ, Liebeskind JC, Albe-Fessard DG, editors. Advances in Pain Research and Therapy. Ely, NY: Raven Press (1979) 3:889–95.

[30] Wercberger R Basbaum AI. Spinal cord projection neurons: a superficial, and also deep, analysis. Curr Opin Physiol. (2019) 11:109–15. 10.1016/j.cophys.2019.10.002 32864531 PMC7450810

[31] Westlund KN. Visceral nociception. Curr Rev Pain. (2000) 4:478–87. 10.1007/s11916-000-0072-9 11060594 PMC7879461

[32] Nathan PW. Results of antero-lateral cordotomy for pain in cancer. J Neurol Neurosurg Psychiat. (1963) 26:353–62. 10.1136/jnnp.26.4.353 14043051 PMC495596

[33] Basbaum AI. Conduction of the effects of noxious stimulation by short-fiber multisynaptic systems of the spinal cord in the rat. Exp Neurol. (1973) 40:699–716. 10.1016/0014-4886(73)90105-2 4723852

[34] Karplus JP Kreidl A. Ein Beitrag zur Kenntnis der Schmerleitung im Ruckenmark. Pflugers Arch. (1914) 158:275. 10.1007/BF01703425

[35] Fajersztajn J. Untersuchungen über Degeneration nach doppelten Rückenmarksdurchschneidungen. Neurolog. Zentralbl. (1895) Xixv:339–48.

[36] Woolf CJ. Evidence for a central component of post-injury pain hypersensitivity. Nature. (1983) 306:686–688. 10.1038/306686a0 6656869

[37] Porreca F Ossipov MH Gebhart GF. Chronic pain and medullary descending facilitation. Trends Neurosci. (2002) 25:319–25. 10.1016/S0166-2236(02)02157-4 12086751

[38] Zhuo M. Silent glutamatergic synapses and long-term facilitation in spinal dorsal horn. Prog Brain Res. (2000) 129:101–3 10.1016/S0079-6123(00)29008-0 11098684

[39] Szucs P Luz LL Pinho R Aguiar P Antal Z Tiong SY. Axon diversity of lamina I local-circuit neurons in the lumbar spinal cord. J Comp Neurol. (2013) 521:2719–41. 10.1002/cne.23311 23386329 PMC3738926

